# LC–MS/MS based characterisation and differential expression of proteins in Himalayan snow trout, *Schizothorax labiatus* using LFQ technique

**DOI:** 10.1038/s41598-023-35646-y

**Published:** 2023-06-22

**Authors:** Kousar Jan, Imtiaz Ahmed, Nazir Ahmad Dar, Mohammad Abul Farah, Fatin Raza Khan, Basit Amin Shah, Francesco Fazio

**Affiliations:** 1 Fish Nutrition Research Laboratory, Department of Zoology, University of Kashmir, Hazratbal, Srinagar, Jammu and Kashmir 190 006 India; 2 Department of Biochemistry, University of Kashmir, Hazratbal, Srinagar, 190006 India; 3 Department of Zoology, College of Science, King Saud University, Riyadh, 11451 Saudi Arabia; 4 Department of Biotechnology and Bioinformatics, University of Hyderabad, Hyderabad, India; 5 Department of Biotechnology, University of Kashmir, Hazratbal, Srinagar 190006 India; 6 Department of Veterinary Sciences, Polo Universitario Annunziata, University of Messina, 98168 Messina, Italy

**Keywords:** Ecology, Molecular biology, Zoology

## Abstract

Molecular characterization of fish muscle proteins are nowadays considered as a key component to understand the role of specific proteins involved in various physiological and metabolic processes including their up and down regulation in the organisms. Coldwater fish specimens including snow trouts hold different types of proteins which help them to survive in highly diversified temperatures fluctuating from 0 to 20 °C. So, in current study, the liquid chromatography mass spectrometry using label free quantification technique has been used to investigate the muscle proteome profile of *Schizothorax labiatus*. For proteomic study, two weight groups of *S. labiatus* were taken from river Sindh. The proteomic analysis of group 1 revealed that a total of 235 proteins in male and 238 in female fish were recorded. However, when male and female *S. labiatus* were compared with each other on the basis of spectral count and abundance of peptides by ProteinLynx Global Server software, a total of 14 down-regulated and 22 up-regulated proteins were noted in this group. The highly down-regulated ones included homeodomain protein HoxA2b, retinol-binding protein 4, MHC class II beta chain and proopiomelanocortin while as the highly expressed up-regulated proteins comprised of gonadotropin I beta subunit, NADH dehydrogenase subunit 4, manganese superoxide dismutase, recombinase-activating protein 2, glycosyltransferase, chymotrypsin and cytochrome b. On the other hand, the proteomic characterisation of group 2 of *S. labiatus* revealed that a total of 227 proteins in male and 194 in female fish were recorded. When male and female *S. labiatus* were compared with each other by label free quantification, a total of 20 down-regulated and 18 up-regulated proteins were recorded. The down-regulated protein expression of group 2 comprised hepatic lipase, allograft inflammatory factor-1, NADH dehydrogenase subunit 4 and myostatin 1 while the highly expressed up-regulated proteins included glycogen synthase kinase-3 beta variant 2, glycogen synthase kinase-3 beta variant 5, cholecystokinin, glycogen synthase kinase-3 beta variant 3 and cytochrome b. Significant (*P* < 0.05) difference in the expression of down-regulated and up-regulated proteins was also noted between the two sexes of *S. labiatus* in each group. According to MS analysis, the proteins primarily concerned with the growth, skeletal muscle development and metabolism were down-regulated in river Sindh, which indicates that growth of fish during the season of collection i.e., winter was slow owing to less food availability, gonad development and low metabolic activity. While, the proteins related to immune response of fish were also noted to be down-regulated thereby signifying that the ecosystem has less pollution loads, microbial, pathogenic and anthropogenic activities. It was also found that the proteins involved in glycogen metabolism, reproductive and metabolic processes, particularly lipid metabolism were up-regulated in *S. labiatus*. The significant expression of these proteins may be connected to pre-spawning, gonad development and use of stored food as source of energy. The information generated in this study can be applied to future research aimed at enhancing food traceability, food safety, risk management and authenticity analysis.

## Introduction

The name ‘proteome’ was abbreviated from the words ‘PROTEin’ complement of ‘genOME’, depicting the whole set of proteins expressed in a certain state of an organism or cell population^1,2^. Proteins are responsible for the energy generation, relationships, structure, actions and division of cells, and are recognised as a central and inclusive means of comprehending biological systems^3^. Proteomic analysis is used to evaluate proteins on a vast scale in a specific biological system. This type of research entails not only determining the function and structure of proteins, but also their quantification, intracellular localization, alterations, interactions and abundance^4^. Regardless of the intricacy of the tested protein mixtures, which can range from hundreds to thousands of proteins, proteomics serves as the primary purpose in identifying the maximum number of proteins that are expected to occur in those mixtures^5^. It is also regarded as one of the most capable methods for detecting molecular markers that could indicate the many physiological variations occurring because of stress exposure^6^. Despite the limited use of these techniques in the classical aquaculture practices, recent trends imply the huge power of proteomics for the detection of stress signals in aquaculture^7–12^. It also provides sufficient knowledge about microbial and adaptation mechanisms in response to cold stress environvments^13^ and could be used to investigate the physiological adaptations, evolution and biodiversity of fish that live in harsh temperature environments^14^. Proteomics, a high-throughput approach, can be used to analyse fish welfare and diseases, as well as provide information on the distinct proteins involved in various inflammatory and immunological responses^5,12^. Such technique has also been widely applied in a variety of disciplines in aquaculture, including pathogen identification, disease symptomatology, and histological evaluation for the diagnosis of various diseases in aquaculture^5,15,16^.

Protein profiling of fish is a technique for detecting the characteristics and quantities of various protein sets present in their muscles and helps researchers to better understand the physiology and metabolism of skeletal muscles. Muscle is an important part of whole-body protein metabolism because it serves as the main source of amino acids for maintaining protein synthesis in key tissues and organs. In case of fish, skeletal muscle acts as the primary organ system, accounting for 34–48% of the total body weight and displaying the edible portion^17^. Muscle proteomics can be used to analyse contractile tissues that are undergoing physiological changes as a result of hypoxia or some other environmental stress. Muscle proteins associated with body mass disparity in fish can be evaluated, in addition to finding intact or proteolytic fragments of muscle-specific gene products crucial for fish muscle growth. Fish muscle performance is necessary for the quickly evolving global aquaculture business, specifically in terms of quality and production^18^. In fish, muscle composition, which is largely dependent on the integrity and amount of muscle fibres, has a significant role in determining quality including flexibility, texture and water retention capabilities^19^. A range of factors including fish strain, food, exercise training and temperature all have an impact on the amount of muscle fibres^17,19^. Additionally, the muscle proteomics study also aims to recognize novel proteins that could be used as biomarkers for a variety of things, including fish growth, physiology, flesh quality, food safety and aquatic environmental monitoring^20^. Fish contributes about 40% of the total protein taken by approximately two-third of the world's population, making it a significant component of the daily diet in many nations. Only a small number of fish species have had their physiology studied in any great detail, despite the fact that they are all relevant.

**Figure 1:**
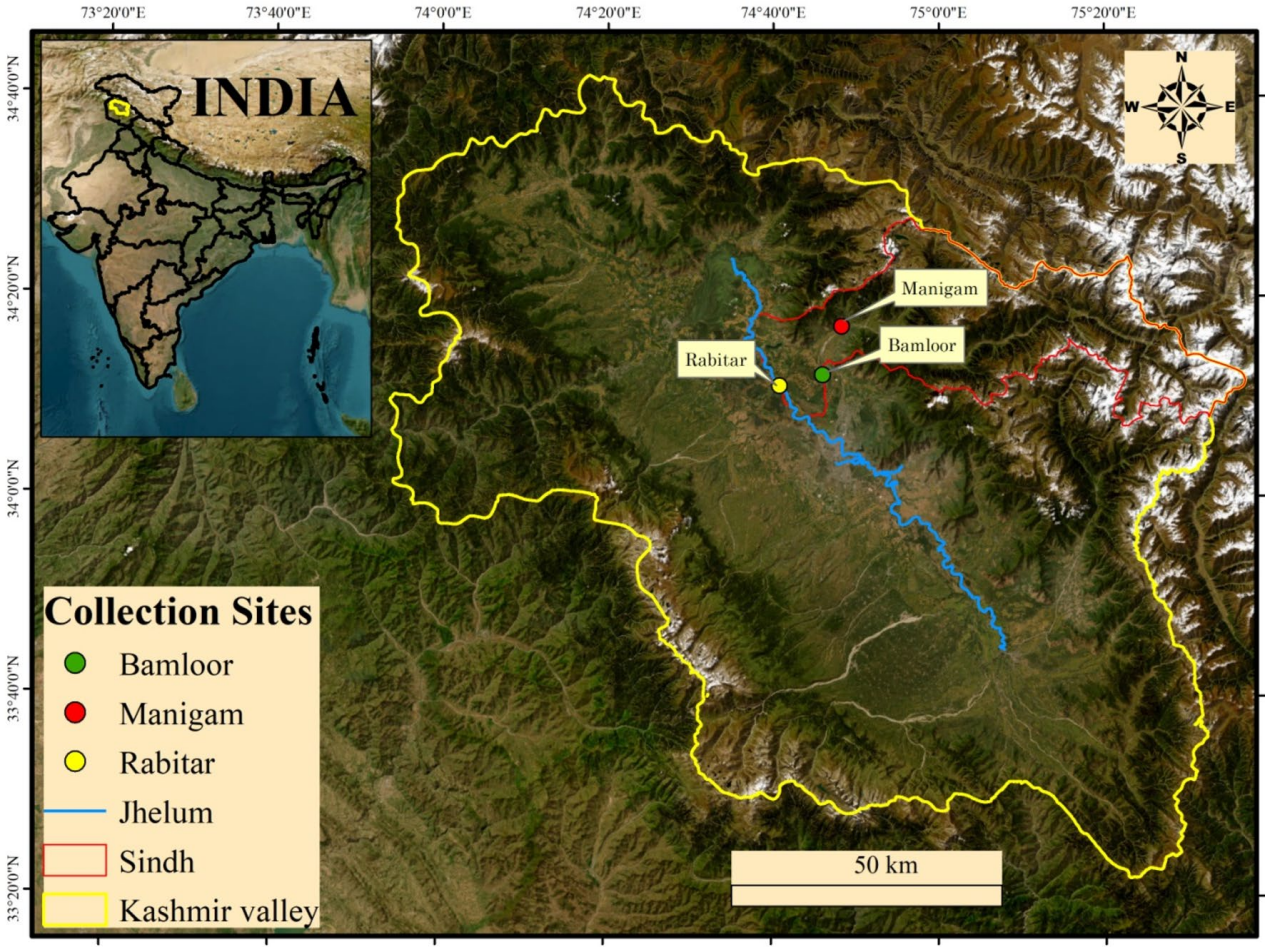
Map of the Kashmir Valley showing locations of the sampling sites along River Sindh (generated in ArcGIS version 10.4.1 by using the imageries provided by Esri, GeoEye, https://www.esri.com).

Since proteomics has become a potent tool for examining biological systems and their dynamics under various situations, it has been progressively applied in recent years to many issues pertaining to fish biology. Since fish is directly related to human health and nutrition, so it is treated to be a foremost source of animal protein to provide food security, therefore as a result, both the general public and the aquaculture industry have started to pay more attention on the quality of fish flesh^21^. *Schizothorax labiatus*, a freshwater fish, is regarded as a significant food fish species in the area, and the water and food supplies are the two main environmental factors that affect how well it lives. Similar to how muscle is organised, protein quantity and composition mostly define the texture of flesh and there are very few research that have been done to find biomarkers for flesh quality^22^. Till date no work on proteomic characterisation of *Schizothorax* spp. has been done so far, therefore the current investigation would be first and novel work in its nature. Genomic data are required to increase the species usefulness in biomarker discovery investigations, but there is a dearth of such data for *S. labiatus*. We examined the *S. labiatus* muscle proteome and produced functional genomic data in the current work (Fig. 1).

## Results

**Figure 2:**
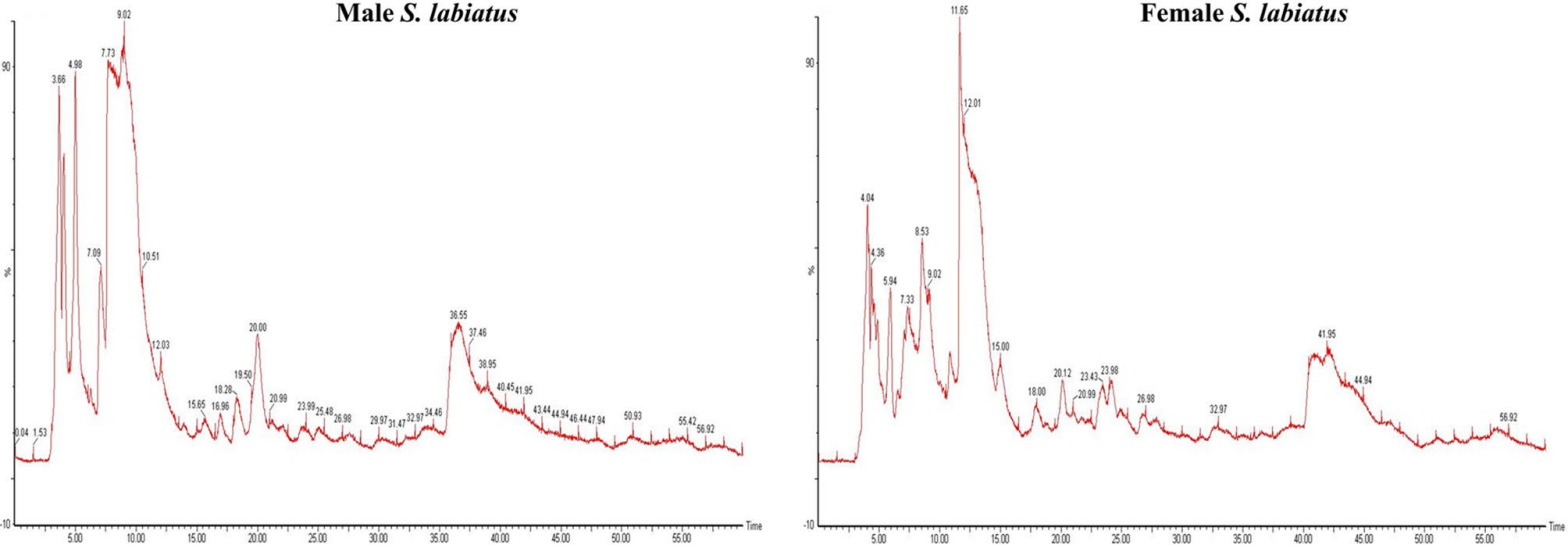
UPLC chromatogram of group 1 male and female *S. labiatus* representing the elution of peptides at different time intervals.

**Figure 3:**
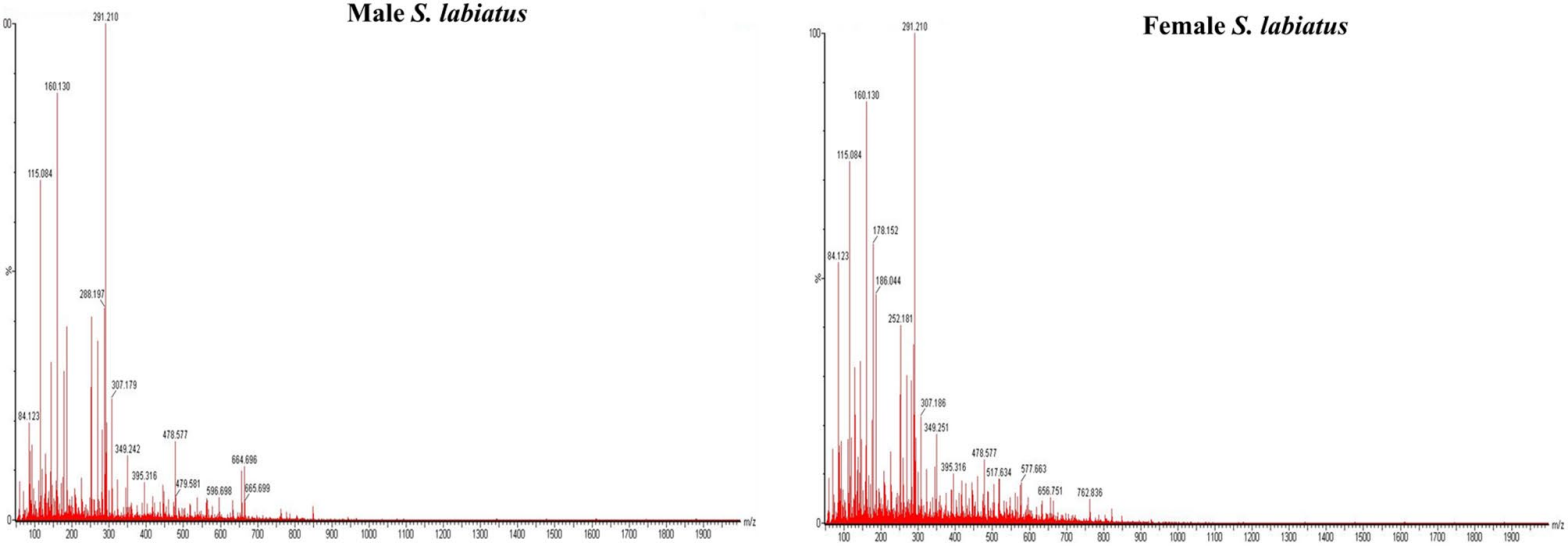
Mass spectrum representing all the eluted peptides of group 1 male and female *S. labiatus.*

The physico-chemical characteristics of the river Sindh and wet laboratory is depicted in Table 1. For proteomic study, two weight groups of *Schizothorax labiatus* were taken from river Sindh. The proteomic analysis of group 1 revealed that a total of 235 proteins in male and 238 in female fish were recorded. The UPLC chromatogram representing the elution of peptides at different time intervals of group 1 male and female *S. labiatus* is mentioned in Fig. 2 and the mass spectrum depicting all the eluted peptides m/z of group 1 male and female is given in Fig. 3. Label free quantification (LFQ) which is a relative quantification method to quantify the differential expression of proteins has been used. However, when male and female *S. labiatus* were compared with each other on the basis of spectral count and abundance of peptides by ProteinLynx Global Server software (PLGS, Waters Corporation), a total of 14 down-regulated and 22 up-regulated proteins were noted. Significant (*P* < 0.05) difference in the expression of down-regulated and up-regulated proteins was recorded between the two sexes of *S. labiatus*. The remaining portion comprised of differentially expressed proteins in group 1 from river Sindh. The down and up-regulated protein expression of group 1 of *S. labiatus* from river Sindh is presented in Table 2. The highly down-regulated ones included homeodomain protein HoxA2b, retinol-binding protein 4, MHC class II beta chain and proopiomelanocortin. While as the highly expressed up-regulated proteins included gonadotropin I beta subunit, NADH dehydrogenase subunit 4, manganese superoxide dismutase, recombinase-activating protein 2, glycosyltransferase, chymotrypsin and cytochrome b.

**Table 1:** Physico-chemical characteristics of river Sindh and stocking water tank (mean ± SD).

Parameters	Sindh	Stocking water tank
pH	8.01 ± 0.96	7.50 ± 0.65
Temperature (°C)	6.38 ± 0.75	9.14 ± 0.82
Dissolved oxygen (mg L^−1^)	8.55 ± 1.02	7.45 ± 1.10
Free carbon-dioxide (mg L^−1^)	4.92 ± 0.81	10.36 ± 1.74
Total alkalinity (mg L^−1^)	97.24 ± 3.44	108.78 ± 2.95

**Table 2:** Down and up-regulated protein expression of *S. labiatus* habiting in river Sindh of Kashmir Himalayas (Group 1).

Accession	Description	Species	Score	Fold change	P value
**Down-regulated proteins**					
AIZ49463.1	Homeodomain protein HoxA2b, partial	*Schizothorax oconnori*	278.52	−1.97649	0.037629
QSV51997.1	Cytochrome b, partial (mitochondrion)	*Schizothorax progastus*	294.9	−1.88993	0.026254
QXO37026.1	Retinol-binding protein 4	*Schizothorax prenanti*	299.38	−1.86108	0.022505
AKN79604.1	MHC class II beta chain	*Schizothorax prenanti*	169.05	−1.61582	0.028262
AFT92021.1	Proopiomelanocortin	*Schizothorax prenanti*	361.29	−1.5004	0.01651
AEX86942.1	Beta-actin, partial	*Schizothorax prenanti*	233.2	−1.37056	0.019366
AFU07561.1	Hypoxia-inducible factor 1 alpha subunit	*Schizothorax prenanti*	196.85	−1.16858	0.020493
QOD42481.1	Nucleotide-binding oligomerization domain 2 splicing variant type 2	*Schizothorax prenanti*	149.35	−1.16858	0.045495
ADZ56746.1	Early growth response 3, partial	*Schizothorax davidi*	277.18	−1.15416	0.019898
AJG42182.1	Hypoxia-inducible factor 1 alpha B	*Schizothorax prenanti*	197.15	−1.15416	0.018387
ACF22669.1	Rodopsin	*Schizothorax waltoni*	224.98	−1.09645	0.02891
ALX39691.1	Interphotoreceptor retinoid-binding protein, partial	*Schizothorax prenanti*	88.69	−1.09645	0.030134
YP_010139254.1	Cytochrome c oxidase subunit II (mitochondrion)	*Schizothorax sinensis*	99.45	−1.02431	0.024385
ALX39407.1	Ectodermal-neural cortex 1-like protein, partial	*Schizothorax prenanti*	162.01	−1.00989	0.021429
**Up-regulated proteins**					
AIC80877.1	Ribosomal protein S6	*Schizothorax prenanti*	94.29	1.024313	0.006513
ANK78718.1	Cytochrome oxidase subunit 2 (mitochondrion)	*Schizothorax plagiostomus*	68.68	1.024313	0.014221
AUL77366.1	Interferon regulatory factor 2	*Schizothorax lissolabiata*	146.39	1.03874	0.048759
QHH24168.1	NADH dehydrogenase subunit 3 (mitochondrion)	*Schizothorax davidi*	220.45	1.03874	0.008086
QCQ29663.1	Glyceraldehyde phosphate dehydrogenase beta isoform	*Schizothorax oconnori*	82.5	1.067594	0.022802
ACX31826.1	Somatolactin alpha	*Schizothorax prenanti*	128.61	1.082021	0.035643
ACX31824.1	Growth hormone	*Schizothorax prenanti*	240.5	1.096448	0.022747
AEQ19479.1	Recombination activating protein 2, partial	*Schizothorax argentatus*	220	1.096448	0.032292
YP_009058164.1	NADH dehydrogenase subunit 5 (mitochondrion)	*Schizothorax pseudoaksaiensis*	147.1	1.110875	0.038274
AJQ19454.1	Sidkey-like protein, partial	*Schizothorax macropogon*	261.12	1.154156	0.031617
AJQ19464.1	Zinc finger protein PLAGL2, partial	*Schizothorax oconnori*	112.37	1.298426	0.014419
AII21813.1	Corticotropin-releasing hormone	*Schizothorax prenanti*	114.52	1.341706	0.014267
AIP90160.1	Fatty acid synthase, partial	*Schizothorax prenanti*	289.06	1.384987	0.015995
APX55890.1	Recombination activating protein 1, partial	*Schizothorax lissolabiata*	397.94	1.428268	0.033944
AVY53419.1	Growth hormone receptor, partial	*Schizothorax richardsonii*	269.02	1.485976	0.035461
AEB33872.1	Gonadotropin I beta subunit	*Schizothorax prenanti*	245.43	1.673526	0.021923
AJQ17841.1	NADH dehydrogenase subunit 4, partial	*Schizothorax pelzami*	69.46	1.70238	0.041307
QEV87013.1	Manganese superoxide dismutase, partial	*Schizothorax prenanti*	735.5	1.70238	0.005864
ALX39540.1	Glycosyltransferase, partial	*Schizothorax prenanti*	192.03	1.933211	0.021091
AWB11398.1	Chymotrypsin, partial	*Schizothorax richardsonii*	132.96	1.947638	0.043678
QSV52008.1	Cytochrome b, partial (mitochondrion)	*Schizothorax progastus*	101.51	6.76624	0.038805

*Significant at 5% level of significance.

**Figure 4:**
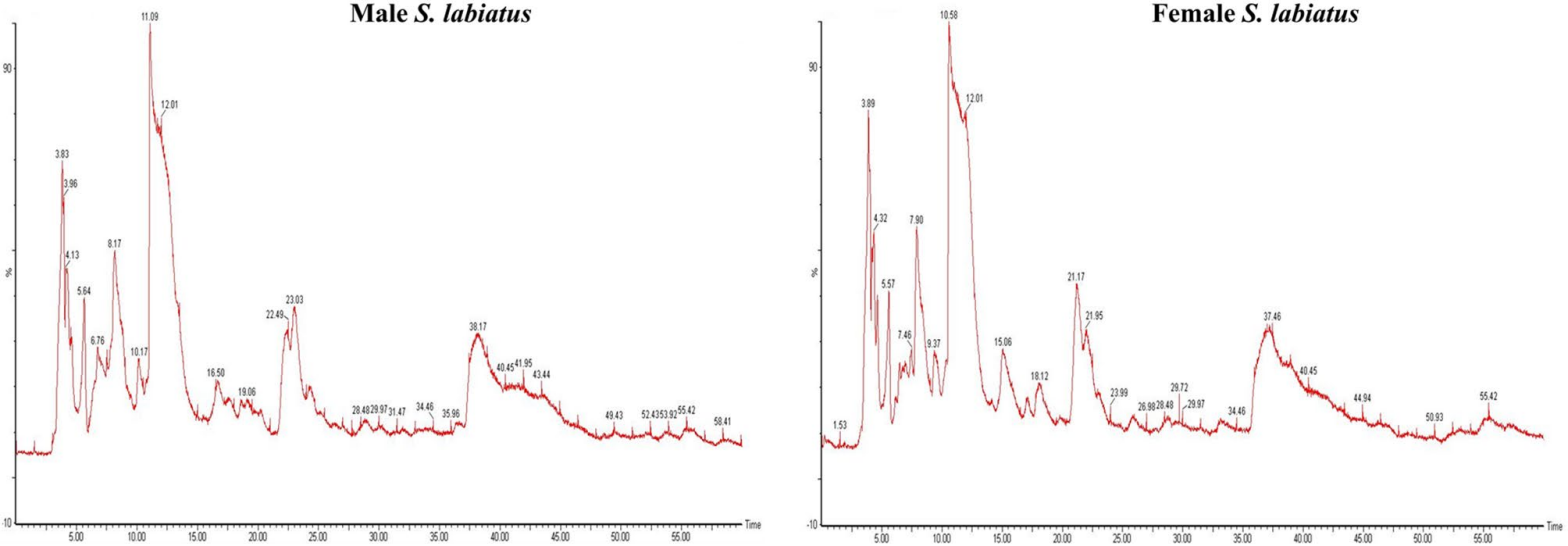
UPLC chromatogram of group 2 male and female *S. labiatus* representing the elution of peptides at different time intervals.

**Figure 5:**
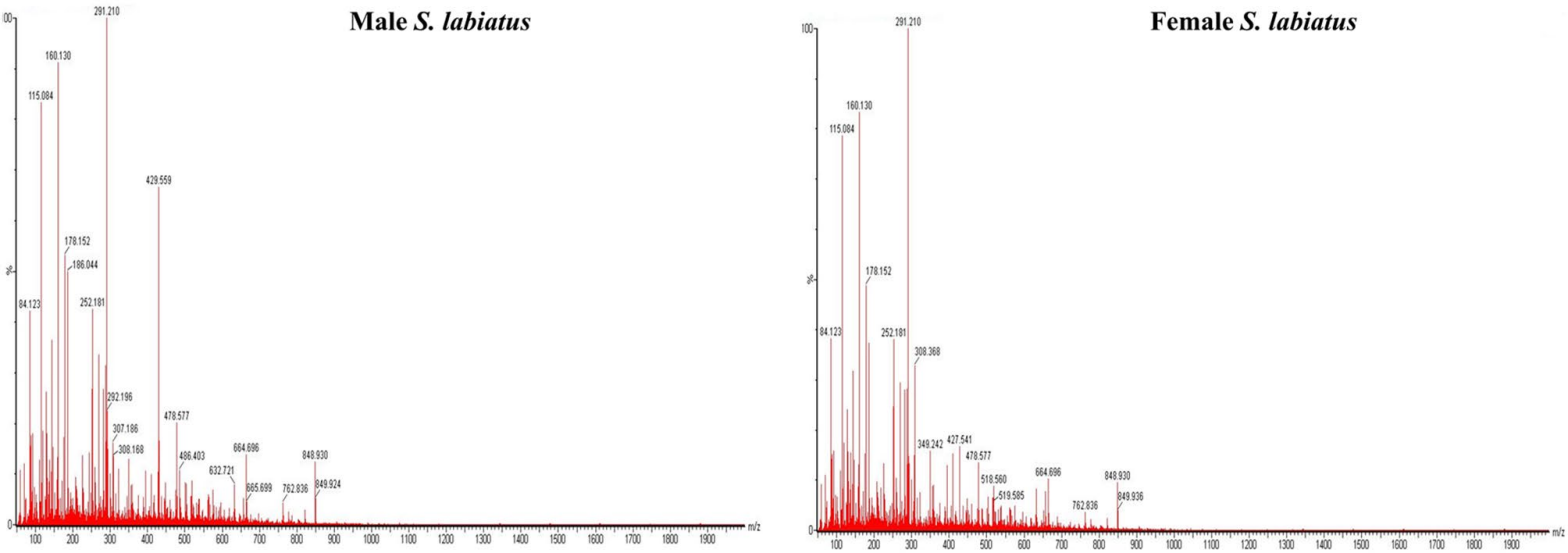
Mass spectrum representing all the eluted peptides of group 2 male and female *S. labiatus.*

On the other hand, the proteomic characterisation of group 2 of *S. labiatus* revealed that a total of 227 proteins in male and 194 in female fish were recorded from river Sindh. The UPLC chromatogram representing the elution of peptides at different time intervals of group 2 male and female *S. labiatus* is depicted in Fig. 4, while the mass spectrum signifying all the eluted peptides m/z of group 2 male and female *S. labiatus* is given in Fig. 5. However, when male and female *S. labiatus* were compared with each other by label free quantification, a total of 20 down-regulated and 18 up-regulated proteins were recorded in this group. The remaining portion comprised of differentially expressed proteins in group 2 of *S. labiatus* from river Sindh. Significant (*P* < 0.05) difference in the expression of down-regulated and up-regulated proteins was also noted between the two sexes of *S. labiatus* under this group. The down and up-regulated protein expression of group 2 of *S. labiatus* is depicted in Table 3. The highly down-regulated ones comprised of hepatic lipase, allograft inflammatory factor-1, NADH dehydrogenase subunit 4 and myostatin 1while as the highly expressed up-regulated proteins included glycogen synthase kinase-3 beta variant 2, glycogen synthase kinase-3 beta variant 5, cholecystokinin, glycogen synthase kinase-3 beta variant 3 and cytochrome b.

**Table 3:** Down and up-regulated protein expression of *S. labiatus* habiting in river Sindh of Kashmir Himalayas (Group 2).

Accession	Description	Species	Score	Fold change	P value
**Down-regulated proteins**					
AWB11400.1	Hepatic lipase, partial	*Schizothorax richardsonii*	124	−2.53914	0.034649
AFA43699.1	Allograft inflammatory factor-1	*Schizothorax prenanti*	324.5	−2.35159	0.047008
AJQ17881.1	NADH dehydrogenase subunit 4, partial (mitochondrion)	*Schizothorax plagiostomus*	138.74	−2.20732	0.015335
QCC30165.1	Myostatin 1	*Schizothorax richardsonii*	453.39	−2.10633	0.01731
AIC80876.1	Ribosomal protein S6 kinase	*Schizothorax prenanti*	126.43	−1.83222	0.035189
AEO15401.1	Recombinase-activating protein 1, partial	*Schizothorax macropogon*	221.25	−1.76009	0.043441
AJQ18139.1	Recombination activating protein 1, partial	*Schizothorax oconnori*	220.22	−1.67353	0.023884
ANW82316.1	Cytochrome P450 aromatase	*Schizothorax kozlovi*	259.77	−1.67353	0.028679
AKC03099.1	Proglucagon	*Schizothorax prenanti*	262.67	−1.58696	0.012465
AEX86942.1	Beta-actin, partial	*Schizothorax prenanti*	589.24	−1.55811	0.012702
AWH12465.1	Fatty acid synthase, partial	*Schizothorax richardsonii*	392.45	−1.42827	0.007018
ALZ45387.1	Erythropoietin receptor	*Schizothorax gongshanensis*	160.32	−1.37056	0.022013
AUL77455.1	Interferon regulatory factor 9	*Schizothorax macropogon*	232.84	−1.37056	0.027072
AUL77358.1	Interferon regulatory factor 2	*Schizothorax lantsangensis*	117.28	−1.34171	0.042758
ANK78582.1	NADH dehydrogenase subunit 5 (mitochondrion)	*Schizothorax waltoni*	241.01	−1.25514	0.035294
AUL77405.1	Interferon regulatory factor 6	*Schizothorax lissolabiata*	214.04	−1.21186	0.00756
AQV11483.1	Aromatse brain type, partial	*Schizothorax richardsonii*	359.3	−1.16858	0.026337
QSV52027.1	Cytochrome b, partial (mitochondrion)	*Schizothorax richardsonii*	216.56	−1.09645	0.026135
ANC60320.1	Ectodysplasin-A	*Schizothorax molesworthi*	188.39	−1.03874	0.0361
QOD42481.1	Nucleotide-binding oligomerization domain 2 splicing variant type 2	*Schizothorax prenanti*	78.32	−1.02431	0.04271
**Up-regulated proteins**					
ANC60315.1	Ectodysplasin-A	*Schizothorax plagiostomus*	171.07	1.03874	0.039759
ACX31824.1	Growth hormone	*Schizothorax prenanti*	486.85	1.082021	0.044498
ADA55403.1	Interphotoreceptor retinoid-binding protein, partial	*Schizothorax waltoni*	198.21	1.082021	0.03035
AJQ19433.1	Ubiquitin-protein ligase E3A, partial	*Schizothorax wangchiachii*	267.71	1.096448	0.049922
QEE82337.1	Elongation factor 1-alpha	*Schizothorax prenanti*	221.94	1.110875	0.005058
ALD15937.1	Cytochrome c oxidase subunit 1 (mitochondrion)	*Schizothorax plagiostomus*	203.15	1.139729	0.036793
BAV71831.1	NADH dehydrogenase subunit 4 (mitochondrion)	*Schizothorax plagiostomus*	103.98	1.154156	0.015849
ANK78578.1	NADH dehydrogenase subunit 2 (mitochondrion)	*Schizothorax waltoni*	116.52	1.197437	0.048909
QWA14834.1	Stearoyl-CoA desaturase 2	*Schizothorax prenanti*	236.21	1.269572	0.014645
AKA09693.1	Glycogen synthase kinase-3 beta variant 1	*Schizothorax prenanti*	253.17	1.500403	0.019044
AEV45801.1	Preproghrelin	*Schizothorax davidi*	192.02	1.572538	0.032504
AKA09696.1	Glycogen synthase kinase-3 beta variant 4	*Schizothorax prenanti*	131.14	1.586965	0.03423
AWB11399.1	Ghrelin	*Schizothorax richardsonii*	192.02	1.659099	0.020499
AKA09694.1	Glycogen synthase kinase-3 beta variant 2	*Schizothorax prenanti*	124.19	1.731234	0.049611
AKA09697.1	Glycogen synthase kinase-3 beta variant 5	*Schizothorax prenanti*	138.28	1.760088	0.008773
AYH63707.1	Cholecystokinin	*Schizothorax davidi*	85.19	1.947638	0.000882
AKA09695.1	Glycogen synthase kinase-3 beta variant 3	*Schizothorax prenanti*	122.5	2.048627	0.012741
QSV52070.1	Cytochrome b, partial (mitochondrion)	*Schizothorax macrophthalmus*	65.93	2.510289	0.006707

*Significant at 5% level of significance.

## Discussion

Over the past ten years, the use of proteomic technology in fish biology has grown significantly^23^. The proteomics has mostly been utilized to study the physiology, nutrition, health, food quality and safety, development biology and influence of pollutants in fish model organisms as well as some commercial aquaculture species, particularly salmonids and cyprinids. It is an unbiased, technology-driven strategy for cataloguing complete protein complements, and is a perfect analytical tool for finding protein changes in health and illness at a high throughput. A proteome map displays the fish species overall fingerprinting and has the potential to uncover novel proteins that might be used as biomarkers for a variety of aquaculture applications, including fish physiology, growth, meat quality, food safety and aquatic environmental monitoring. During the current study, two different weight groups (Group 1 and 2) of male and female *Schizothorax labiatus* were studied from river Sindh. The proteomic characterisation of group 1 of *S. labiatus* demonstrated that when male and female fish were compared with each other, a total of 14 down-regulated proteins were recorded. Among them, there were some of the proteins that were found to be involved in basic physiological processes such as cytochrome b. The cytochrome b gene, which codes for proteins, is necessary for the proper operation of mitochondria and has roughly a thousand base pair length. Among many other mitochondrial genes, the cytochrome b gene has widely been used to study genetic variation^24^, phylogenetic relationships^25^, biogeographical patterns^26^ and taxonomy^27^ in case of many fishes and higher vertebrates. Its nucleotide sequence is species-specific and is chiefly employed in forensic analysis and evolutionary studies^28^. The cytochrome b gene’s rate of evolution is appropriate for examining events that have occurred within the last 20 million years, such as the evolution of the family Cyprinidae where this gene has been used for the study of phylogenetic association in order to place these species at their respective ranks and to determine their biogeography^29^. The relationship of *S. plagiostomus* with other Schizothoracine fishes has been assessed using the cytochrome b gene of 23 closely related species^30^. Based on reconstructed phylogeny in the Qinghai-Tibet Plateau, Qi et al.^31^ employed cytochrome b sequencing data to examine genetic variation and clarify connections among five species of the highly specialised Schizothoracine fishes and three species of the primitive Schizothoracine fishes. A similar function of protein has been reported by a number of workers who carried out their work on different fish species in the past^32–35^. Besides proteins involved in physiological processes, the most down-regulated ones from river Sindh included homeodomain protein HoxA2b, retinol-binding protein 4, MHC class II beta chain and proopiomelanocortin.

Hox proteins, also known as homeobox proteins, are transcription factors that are crucial for embryonic development in all species. By regionalizing the antero-posterior (A-P) axis and establishing different body plans throughout the animal kingdom, hox genes play a significant role in directing developmental pathways among all bilaterian animals. Hox genes are located in clusters in teleosts that range in size from 5 to 8 (Aa, Ab, Ba, Bb, Ca, Cb, Da, Db) and are divided into 13 paralog groups (PG)^36^. The well-conserved homeobox or homeodomain found in hox genes is assumed to be involved in DNA binding and transcriptional activation^37^. The development of numerous morphological traits depends on hox genes, which encode transcription factors. Recent research has demonstrated that genome replication, sequence variation, and selective pressure were all significantly influenced by the inception and evolution of the Hox genes^38^. Although, it has been extremely rare for animals to experience genome duplications, some vertebrate lineages, like fish have experienced them more frequently than others^39^. The short term impact of genome duplications is still not well understood. Salmonids, sturgeons, and cyprinids are among the unrelated fish taxa that exhibit polyploidy, which may have had a significant impact on the evolution of regulatory mechanisms^40^. According to Li & Guo^41^, the Cyprinidae family contains 400 closely related polyploid species that have either undergone auto or allo-polyploid evolution. There is a significant discrepancy between conventional and molecular classifications within the Cyprinidae. In contrast to the traditional classification of cyprinids into ten subfamilies, 12 subfamilies have been recognised in China^42^. Species in one subfamily are dispersed among numerous subfamilies as per previous molecular phylogenies. Ten traditional subfamilies, whose species have hybrid origins were rejected by the topologies because they failed to resolve monophyletic relationships^43,44^. The Cyprinidae’s history of polyploidization is yet unknown, which presents substantial difficulties for phylogenetic systematics. The timing of complete genome duplications, the sources of polyploidization and other issues are uncertain. HOX genes and gene-copies from 14 representative species, one species complex, including seven subfamilies and one genus of Cyprinidae have been used to shed light on the extensive history of polyploidization^45^. In a homodiploid fish lineage resembling crucian carp, the fast variation of Hox clusters shows a distinct evolutionary path^46^. Analogous work has been reported by a number of workers who carried out their work on different fish species^47–49^. The family of proteins known as retinol-binding proteins (RBP) are a group of carrier proteins that bind retinol and have a variety of functions. The 21 KDa monomeric fish RBP protein has a single retinol binding site^50^. Retinol, popularly known as vitamin A, serve as crucial signalling molecule for regulation during embryogenesis and is indispensable for growth, vision, reproduction, hematopoiesis, immunological function and epithelial cell maintenance in adult animals. RBP works as a transport protein for plasma and cellular retinoids as well as a transporter of tiny hydrophobic molecules like lipids, steroids and other substances to the target tissues in vertebrates^51^. Although, a similar kind of work has been reported by some researchers in the past^52–54^.

The other down-regulated protein in *S. labiatus* included MHC II molecules, also known as major histocompatibility complex class II, which are essential for the adaptive immune response. Regarding gene architecture, polymorphism, protein structure and composition, fish MHC II is comparable to its mammalian counterparts. Juul-Madsen et al.^55^ discovered that MHC II gene in rainbow trout was solely expressed in the spleen and pronephros and was considerably down-regulated after acute viral infection^56^. Red seabream’s MHC II gene expression was likewise lowered after *Vibrio anguillarum* infection with pathogenic bacteria^57^. In contrast, the up-regulation of MHC II gene expression has been recognized in Atlantic salmon^58^, Nile tilapia^59^, blunt nose black bream^60^ and lined seahorse^61^ after immunization with bacterial vaccine. These studies showed how challenging it has been to regulate the expression of the fish MHC II gene. Numerous separate workers have completed a substantial quantity of work that is comparable^62–66^. A precursor to endorphin, melanocyte-stimulating hormone and adrenocorticotropic hormone, is proopiomelanocortin (POMC). It is a crucial precursor for a number of peptide hormones involved in a wide range of processes, including the stress response and energy homeostasis^67^. The POMC-derived peptide MSH interacts with melanocortin-4 receptors in mammals and fish, where it is known to have a role in appetite regulation^68^. Considerable work has been carried out by several workers who performed their studies on different fish species^69–74^. The homeodomain and retinol-binding proteins which are primarily concerned with the growth of *S. labiatus* were noted to be down-regulated in river Sindh which could be linked to the fact that fishes were collected during winter season and availability of food was less. Moreover, it could be also associated with the pre-spawning period of the fish during winter season. A similar down-regulated expression of these proteins in response to cold temperature and reproductive activity was demonstrated by a number of workers^75–77^. The proteins MHC class II beta chain and proopiomelanocortin which are primarily concerned with the immune and stress response were also noted to be down-regulated in river Sindh, indicating that the ecosystem has cold temperature, less pathogenic activity and pollution loads. An analogous down-regulation of these proteins in response to decreased temperature, stress, pathogenic activity, etc. has also been observed by a number of workers in the past^78–81^.

During the current study, it was observed that on comparing male and female *S. labiatus* of group 1, a total of 22 up-regulated proteins were recorded from river Sindh. The highly expressed up-regulated proteins included gonadotropin I beta subunit, NADH dehydrogenase subunit 4, manganese superoxide dismutase, glycosyltransferase, chymotrypsin and cytochrome b. The study of gonadotropins in teleosts has been complicated, and our understanding of how they work is still limited^82^. Despite the fact that for a quite long time, it was thought that fish gonadal growth and function were controlled by a single gonadotropin^83^. In 1988, two chemically different gonadotropins, known as GTH-I and GTH-II were described in the chum salmon, *Oncorhynchus keta*, defining the idea of two gonadotropins that had been hypothesised subsequently after the mid 1970s on the basis of histochemical and biochemical evidence^84^. Since then, various teleost research have shown the dual nature of gonadotropins, and the structural cum functional studies have led to the suggestion that GTH-I and GTH-II should now be known as FSH and LH^85^. The current consensus is that fish FSH, like its human counterparts, primarily promotes early gonadal growth and development, while fish LH is crucial for controlling the final stages of gametogenesis, including the gamete maturation and release^84^. Similar function of protein has been reported in different fish species by a number of workers^86–90^. The antioxidant metalloenzyme, manganese superoxide dismutase (MnSOD) is nuclear-encoded. This enzyme’s primary role in fish is to convert hazardous by-products such as superoxide anion into less harmful hydrogen peroxide and oxygen^91^. The levels of hazardous metabolites, including 64 reactive oxygen species (ROS) and reactive oxygen intermediates (ROI), rise as a result of oxidative stress in fish^92^. Increased oxygen intake during phagocytosis causes respiratory burst while producing ROS. Therefore, by destroying invasive bacteria and hazardous substances, these ROS play a crucial function in the defence mechanism of cells; however, excessive ROS can harm fish internal organs and prevent them from surviving by causing cellular damage, both of which may result in fish death^93^. Thus, by transforming ROS into safe molecules, these antioxidant enzymes are crucial for their control^91^. Many researchers conducted analogous work on various fish species^94–96^.

The other up-regulated protein in *S. labiatus* included a functional class of intracellular membrane bound enzymes known as glycosyltransferases. They cooperate in the manufacture of glycoprotein and glycolipid carbohydrate moieties, which are crucial for a variety of cellular processes^97^. Glycosyltransferase has been discovered to catalyse the transport of sugar moieties from active donor molecules to specific acceptor molecules, resulting in glycosidic connections, even if the acceptor molecule can be a protein, lipid, heterocyclic chemical or any other carbohydrate residue^98^. Few investigations on similar protein have been reported by some researchers^97,99^. Besides this, the other up-regulated protein expressed included endopeptidase digesting enzyme chymotrypsin. The two main types of fish chymotrypsin are typically anionic (chymotrypsin A) and cationic (chymotrypsin B)^100^. Fish chymotrypsins are similar in molecular weight and amino acid content to mammalian chymotrypsin. These have lower pH tolerance and higher specific activity, especially those from coldwater fish^101^. Fish species, age, weight and malnutrition are among the factors that affect the concentration and activity of chymotrypsin in fish. Extensive work on function of protein has been reported by number of workers^102–106^. The findings made it abundantly clear that *S. labiatus* in river Sindh had highly expressed proteins involved in reproductive and metabolic processes, particularly lipid metabolism especially gonadotropin I beta subunit and glycosyltransferase. These proteins may be connected to fish pre-spawning, gonad development and use of stored food as a source of energy. Rocha et al.^107^ also reported a similar up-regulated expression of proteins during various reproductive activities of fishes.

The proteomic characterisation of group 2 of *S. labiatus* clearly depicted that a total of 227 proteins in male and 194 in female fish were recorded. However, when male and female fish were compared with each other, a total of 20 down-regulated proteins were recorded. Among them the most down-regulated ones comprised hepatic lipase, allograft inflammatory factor-1, NADH dehydrogenase subunit 4 and myostatin 1. The liver produces and secretes hepatic lipase (HL), which is a key gene in lipid metabolism and is essential for fish growth^108^. It is a member of the family of lipase genes and is crucial for lipid metabolism^109^. An excessive amount of lipids in the feed may lead to fat accumulation, which will harm the fish. The expression of HL may be influenced by a number of variables, such as hormones, fasting, feeding circumstances, nutritional status, water temperature, and season. Several researchers, including those listed below have been acknowledged for doing extensive study on the similar function of protein^110–116^. The protein known as allograft inflammatory factor-1 (AIF-1) has been extensively researched in vertebrates, particularly in mammals. This element, which is typically engaged in inflammatory reactions to pathogenic infection or tissue damage, is connected to a number of serious illnesses^117^. AIF-1 is a 17 kDa protein that responds to inflammation and is mostly generated by immunocytes^118^. AIF-1, a pro-inflammatory cytokine, regulates the immune system at multiple crucial sites and participates as a critical modulator in inflammatory disease processes. The protein has also been found in oyster, sea cucumber, scallop *Chlamys farreri* and *Crassostrea gigas* as a pro-inflammatory factor linked to immunological response, although in freshwater fish, the inflammatory properties of AIF-1 still haven’t been evidently elucidated^119^. Few studies of allograft inflammatory factor research have been done on various fish species^120–122^. Growth and differentiation factor called myostatin (MSTN) is a key regulator of skeletal muscle growth and development. A recent evolutionary investigation showed that the teleost fish has at least two MSTN genes, MSTN-1 and MSTN-2, which were articulated in both muscular and non-muscular tissues^123^. A lot of researchers have done an exhaustive work on the similar occurrence of protein in various fish species^124–128^. The results signified that in river Sindh, the proteins related to growth, skeletal muscle development and metabolism were down-regulated which indicates that the growth of *S. labiatus* during the season of fish collection i.e., winter was slow owing to less food availability, gonad development and low metabolic activity. Moreover, the proteins related to immune response of fish such as allograft inflammatory factor and myostatin were noted to be down-regulated thereby signifying the less microbial, pathogenic and anthropogenic activities in river Sindh.

In the current investigation, a total of 18 up-regulated proteins were recorded from river Sindh on comparing male and female *S. labiatus* of group 2. The highest up-regulated proteins included glycogen synthase kinase-3 beta variant 2, glycogen synthase kinase-3 beta variant 5, cholecystokinin, glycogen synthase kinase-3 beta variant 3 and cytochrome b. Several cellular functions, including protein synthesis, glycogen consumption, mitosis and apoptosis depend on the enzyme i.e., glycogen synthase kinase-3 (GSK-3)^129^. It is a serine/threonine kinase encoded by the GSK3α and GSK3β genes^130^. Previous studies have shown that GSK3β is made up of two distinct isoforms: GSK3-1, which is distributed across many organs, and GSK3-2, which is exclusively found in the central nervous system^131^. In zebrafish, GSK3α and GSK3β have distinct functions during embryonic cardiogenesis. Few investigations on GSK have been conducted in fish, while the majority have been done on mammals^132–136^. The gastro-intestinal tract along with the central and peripheral neurological systems, is extensively distributed with the peptide hormone cholecystokinin (CCK) in both mammalian as well as non-mammalian species. Specific endocrine cells dispersed across the intestinal mucosa of the digestive organs produce CCK, which is then post-translationally cleaved by enzymes to produce physiologically dynamic CCK/gastrin-like peptides^137^. By promoting the release of pancreatic enzymes like trypsin and chymotrypsin through gut motility and gallbladder contraction, the protein CCK has been shown to play a crucial role in the digestive processes of vertebrates together with teleosts^138^. There are just two studies that illustrate the function of CCK receptors: one is in goldfish^139^ and the other one for Siberian sturgeon^140^. A number of researchers that worked on various fish species have revealed that proteins have similar functions^141–146^. The current study’s findings demonstrate unequivocally that in the river Sindh, proteins involved in glycogen metabolism were significantly elevated, leading to an increase in glycogen metabolism in response to fish activity prior to spawning.

Proteomics has just recently been used to the biology and aquaculture of fish. However, the studies that have been done so far have shown that proteomics has the ability to uncover physiologically appropriate molecules, mechanisms and biomarkers for fish welfare and to assess the effects of environmental contamination. The LCMS proteome profile of different size groups of *Schizothorax labiatus* was carried out in the current study and diverse proteins included in diverse biological functions and processes were discovered. The findings might serve as a starting basis for the *S. labiatus* muscle proteome database. The completed work may potentially lay the foundation for future research aiming at creating novel molecular techniques and protocols for tracking growth dynamics, traceability and quality control of wild *S. labiatus*. The proteome map of the *Schizothorax* spp. needs to be understood more on seasonal basis in order to establish them as suitable models for muscle and other developmental investigations.

## Materials and methods

### Study area

The river Sindh, which originates in the Indian state of Jammu and Kashmir’s Ganderbal district, has a length of around 108 kms and is considered to be a significant tributary of the river Jhelum. The Machoi Glacier, which is situated east of Amarnath Temple and south of the Zojila Pass between 34° 12′ 14.860′′ N and 75° 35′ 21.94′′ E at an elevation of 4800 m (15,700 feet), is the main water source of the river. The river is mostly flowing west the length of National Highway 1D, supplemented by plentiful glacier tributaries, till it meets the river Jhelum at Shadipora Srinagar. For carrying out the current study, three sites were selected from river Sindh i.e., Site I Manigam** (**34° 16ʹ 36.15ʺ; 74° 48ʹ 31.45ʺ), Site II Bamloor (34° 12ʹ 08.23ʺ; 74° 46ʹ 04.38ʺ) and Site III Rabitar (34° 11ʹ 01.70ʺ; 74° 40ʹ 52.15ʺ) (Fig. 1).

### Collection and identification of fish specimens

Live and healthy samples with no perceptible signs of infestations, injuries or deformities on the external body surface of male and female *Schizothorax labiatus* of two different weight and size groups were collected from river Sindh by means of cast nets with the assistance of local fishermen. The first group comprised of 255.42 ± 4.06 g, 28.65 ± 1.12 cm of male and 260.18 ± 5.64 g, 29.24 ± 1.38 cm of female, while as the second group comprised of 420.18 ± 8.44 g, 35.24 ± 1.56 cm of male and 414.53 ± 7.26 g, 34.76 ± 1.90 cm of female *S. labiatus*. After being collected, the fish were recognized using the standard keys provided by Kullander et al.^147^, while sex differentiation was primarily accomplished by examining physical traits like body size and form, colour, dorsal fin spine and caudal fin lobe shapes, snout shape and anal fin length^148–150^. Also the male and female fish can easily be differentiated in the breeding season due to the presence of spilling eggs and milt when the belly was lightly pressed. Additionally, during the breeding season, the males also developed prominent, pointed, whitish tubercles on their heads and other areas of body^151^. The fish were then transferred in open tanks to research stations with the same water as the capture region and were initially immersed in a KMnO_4_ solution to combat off any microbial infections. After that, they were kept for two hour acclimation in well-aerated circular fish tanks containing 70 L of water and had a mechanism that allowed water to flow continuously (1.5–2.5 L min^−1^) in order to ease their stress from transit, netting, handling, etc.

### Protein profiling

For proteomic study i.e., verifying the presence of species-specific proteins in the muscles of fishes, live specimens of different sizes and weights of male and female *S. labiatus* were placed in a tank and euthanized with an overdose of tricaine methanesulfonate (MS-222) on the day of tissue sampling^152^. Skeletal muscle samples were taken between the head and dorsal fin from the epaxial musculature dorsal to the lateral line. The muscle tissue of the fish was then divided into smaller pieces and stored at −80 °C in sterile universal bottles prior to use. Three times with chilled phosphate buffered saline (PBS), the frozen tissues were rinsed. With a pre-cooled mortar and pestle, 200 mg of tissue was pulverised to powder in liquid nitrogen. The powder was placed in lysis buffer on ice for 30 min. The resultant lysates were vortexed and incubated for 10 min at room temperature. After centrifugation at 12,000 rpm at 4 °C to remove particulate debris, the protein solutions were collected and kept at −80 °C until usage. From the stored protein solutions, 100 μg of the sample was taken for the purpose of digestion. The sample was then diluted with 50 mM ammonium bicarbonate (NH_4_HCO_3_). After that, the sample was treated with 100 mM dithiothreitol (DTT) at 95 °C for 1 h followed by 250 mM iodoacetamide (IDA) at room temperature in dark for 45 min. The sample was next subjected to digestion with trypsin where 20 μg of trypsin vial (Promega) was dissolved in 100 μL of ammonium bicarbonate and 2  μL of this solution was used for every 100 μg of protein sample. Overnight incubation was performed on the sample at 37 °C. The resultant sample was vaccum dried and then dissolved in 20  μL of 0.1% formic acid in water. The supernatant was collected into a separate tube, after centrifugation at 10,000 g. Then 10 µL injection volume was used on BEH C18 UPLC (ethylene bridged hybrid C18 ultra performance liquid chromatography) column for separation of peptides. After the separation of peptides on the column, the peptides were directed to Waters Synapt G2 Q-TOF instrument for MS and MSMS analysis (Tandem mass spectrometry). The raw data was evaluated by MassLynx 4.1 WATERS and the individual peptides MSMS spectra were matched to the database sequence for recognition of proteins on PLGS software, WATERS.

### Physico-chemical properties

For the investigation of physico-chemical characteristics, water samples were taken from all the chosen study locations of the river Sindh. The physico-chemical characteristics of stocking tanks were also recorded in the wet laboratory. Using established techniques, the water parameters i.e., pH, water temperature, dissolved oxygen, free carbon dioxide and total alkalinity were determined^153^.

### Statistical analysis

The data representation was carried out by using the Microsoft Excel 2013. The protein abundance among male and female *S. labiatus* was determined statistically using R programming language^154^. To satisfy the normal distribution and homogeneity of variance assumptions, data was transformed using either the natural logarithm or the square root. One way ANOVA was used to compare the differences in protein abundance between two sexes of *S. labiatus*. To determine the significance of the pair wise comparisons for those proteins, the Tukey’s post-hoc analysis was used to evaluate the p value. Adjusted p-values below the significance threshold of = 0.05 were required for differences to be deemed meaningful. If the absolute fold change was at least 1 and the adjusted p value was below (fold change 1 or >  + 1), then protein abundance was deemed to be significant.

### Label-free quantification

The LC-MS^E^ data was used to identify and quantify the proteins in the mixture using a proven label-free quantification technique (Hi-3 method) developed by Silva et al.^155^. The concentration of a protein in a complex mixture has been estimated using the added electrospray ionisation mass spectrometry (ESI–MS) signal of the top three best responding peptide precursors^155–157^. 3–6 technical replicate of each biological sample in LCMS were injected and the three best results were chosen for further analysis. The *S. labiatus* proteins were downloaded from Uniprot and a protein database was created on PLGS software to start the analysis. The three best technical triplicates were ran against the prepared database and prepared as group for biological replicates. An expression analysis embedded tools of PLGS software has been used which finds the differential expression of proteins between the groups such as control and treated samples. In our case, we used *S. labiatus* male and female groups to find the expression. The most abundant peptides of all proteins detected in LC–MS dataset were compared with the three most intense peptides of other group samples.

### Ethical approval

During the present research work, all applicable international, national, and/or institutional guidelines for the care and use of animals were followed. All the protocols used have been approved by Animal Ethical Committee registered under Committee for the Purpose of Control and Supervision of Experiments on Animals (CPCSEA), Animal Experimentation and Ethics in India, 2018 with R. No. 801/Go/RE/S/2003/CPCSEA.

### Compliance with the ARRIVE guidelines

All the methods used in the study were carried out in accordance with ARRIVE guidelines.

## Consent for publication

All authors examined the manuscript and gave their approval for publishing.

## Data Availability

Upon request, the corresponding author will provide access to the data that underpin all of this study’s analysis.
