# Author Correction: LC–MS/MS based characterisation and differential expression of proteins in Himalayan snow trout, *Schizothorax labiatus* using LFQ technique

**DOI:** 10.1038/s41598-023-38786-3

**Published:** 2023-07-18

**Authors:** Kousar Jan, Imtiaz Ahmed, Nazir Ahmad Dar, Mohammad Abul Farah, Fatin Raza Khan, Basit Amin Shah, Francesco Fazio

**Affiliations:** 1 Fish Nutrition Research Laboratory, Department of Zoology, University of Kashmir, Hazratbal, Srinagar, Jammu and Kashmir 190 006 India; 2 Department of Biochemistry, University of Kashmir, Hazratbal, Srinagar, 190006 India; 3 Department of Zoology, College of Science, King Saud University, Riyadh, 11451 Saudi Arabia; 4 Department of Biotechnology and Bioinformatics, University of Hyderabad, Hyderabad, India; 5 Department of Biotechnology, University of Kashmir, Hazratbal, Srinagar 190006 India; 6 Department of Veterinary Sciences, Polo Universitario Annunziata, University of Messina, 98168 Messina, Italy

Correction to: *Scientific Reports* https://doi.org/10.1038/s41598-023-35646-y, published online 22 June 2023

The original version of this Article contained an error in the Acknowledgements section.

“The authors thank Head, Department of Zoology, University of Kashmir for providing the necessary laboratory services.”

now reads:

“The authors thank Head, Department of Zoology, University of Kashmir for providing the necessary laboratory services. The authors would like to extend their sincere appreciation to the Researchers Supporting Project number (RSPD2023R694), King Saud University, Riyadh, Saudi Arabia.”

In addition, the original version of this Article contained an error in the Funding section.

“The Department of Science and Technology (DST), Government of India, New Delhi is thanked by the authors for providing Kousar Jan with financial assistance in the form of a DST-INSPIRE fellowship. This work was partially supported by the Researchers Supporting. Project number (RSP-2023R1/154), King Saud University, Riyadh, Saudi Arabia.”

now reads:

“The Department of Science and Technology (DST), Government of India, New Delhi is thanked by the authors for providing Kousar Jan with financial assistance in the form of a DST-INSPIRE fellowship. This work was also funded by Researchers Supporting Project number (RSPD2023R694), King Saud University, Riyadh, Saudi Arabia.”

The original Article has been corrected.

